# The spring-like effect of microRNA-31 in balancing inflammatory and regenerative responses in colitis

**DOI:** 10.3389/fmicb.2022.1089729

**Published:** 2022-12-16

**Authors:** Jing Qu, Chunlei Shao, Yongfa Ying, Yuning Wu, Wen Liu, Yuhua Tian, Zhiyong Yin, Xiang Li, Zhengquan Yu, Jianwei Shuai

**Affiliations:** ^1^Department of Physics, and Fujian Provincial Key Laboratory for Soft Functional Materials Research, Xiamen University, Xiamen, China; ^2^State Key Laboratories for Agrobiotechnology, College of Biological Sciences, China Agricultural University, Beijing, China; ^3^Department of Mathematics and Physics, Fujian Jiangxia University, Fuzhou, China; ^4^National Institute for Data Science in Health and Medicine, Xiamen University, Xiamen, China; ^5^State Key Laboratory of Cellular Stress Biology, Innovation Center for Cell Signaling Network, School of Life Sciences, Xiamen University, Xiamen, China; ^6^Oujiang Laboratory (Zhejiang Lab for Regenerative Medicine, Vision and Brain Health), University of Chinese Academy of Sciences, Wenzhou, China; ^7^Wenzhou Institute, Wenzhou Key Laboratory of Biophysics, University of Chinese Academy of Sciences, Wenzhou, China

**Keywords:** MIR31, inflammatory response, epithelial regeneration, spring-like effect, network modeling

## Abstract

Inflammatory bowel diseases (IBDs) are chronic inflammatory disorders caused by the disruption of immune tolerance to the gut microbiota. MicroRNA-31 (MIR31) has been proven to be up-regulated in intestinal tissues from patients with IBDs and colitis-associated neoplasias. While the functional role of MIR31 in colitis and related diseases remain elusive. Combining mathematical modeling and experimental analysis, we systematically explored the regulatory mechanism of MIR31 in inflammatory and epithelial regeneration responses in colitis. Level of MIR31 presents an “adaptation” behavior in dextran sulfate sodium (DSS)-induced colitis, and the similar behavior is also observed for the key cytokines of p65 and STAT3. Simulation analysis predicts MIR31 suppresses the activation of p65 and STAT3 but accelerates the recovery of epithelia in colitis, which are validated by our experimental observations. Further analysis reveals that the number of proliferative epithelial cells, which characterizes the inflammatory process and the recovery of epithelia in colitis, is mainly determined by the inhibition of MIR31 on IL17RA. MIR31 promotes epithelial regeneration in low levels of DSS-induced colitis but inhibits inflammation with high DSS levels, which is dominated by the competition for MIR31 to either inhibit inflammation or promote epithelial regeneration by binding to different targets. The binding probability determines the functional transformation of MIR31, but the functional strength is determined by MIR31 levels. Thus, the role of MIR31 in the inflammatory response can be described as the “spring-like effect,” where DSS, MIR31 action strength, and proliferative epithelial cell number are regarded as external force, intrinsic spring force, and spring length, respectively. Overall, our study uncovers the vital roles of MIR31 in balancing inflammation and the recovery of epithelia in colitis, providing potential clues for the development of therapeutic targets in drug design.

## Introduction

Inflammatory bowel diseases (IBDs), including ulcerative colitis (UC) and Crohn’s disease (CD), are chronic inflammatory disorders that impact gastrointestinal tract (Kaser et al., 2010). Chronic inflammatory disorders are characterized by submucosal accumulation of immune cells, resulting in damage to the epithelial layer (Maloy and Powrie, 2011; Cader and Kaser, 2013). The prevalence and incidence of IBD have continued to increase over the past few decades around the world (Xavier and Podolsky, 2007; Kaplan and Ng, 2017; Ng et al., 2017), but the precise etiology and pathogenesis of IBD have not been fully revealed. Recent studies have shown the significant role of gut microbiota in IBD (Seksik, 2010; Dalal and Chang, 2014; Sheehan et al., 2015). A widely accepted pathogenesis is that environmental or genetic factors trigger an abnormal immune response to the gut microbiota in a genetically susceptible host (Fava and Danese, 2011; Becker et al., 2015; Matsuoka and Kanai, 2015). IBD is associated with marked changes in gene expression and protein level (Neurath, 2014; Kumar et al., 2016). Increasing studies show that microRNAs (miRNAs) play vital roles in the regulation of IBD (Dalal and Kwon, 2010; Kalla et al., 2015; Xu and Zhang, 2016; Soroosh et al., 2018). MiRNAs are a class of small noncoding RNAs with a length of approximately 18–25 nucleotides (Bartel, 2004; Emde and Hornstein, 2014), which are widely found in nematodes, fruit flies, plants and eukaryotes (Bartel, 2009; Carthew and Sontheimer, 2009; Fabian et al., 2010). MiRNAs have been estimated to regulate over 60% of protein-coding genes (Garzon et al., 2010; Lin et al., 2013a; Hammond, 2015; Treiber et al., 2018) by base pairing with target mRNAs and repressing translation (Krol et al., 2010; Ha and Kim, 2014). Abnormal expression of miRNAs is highly correlated with many diseases including cancer (Wu et al., 2008; Schaefer et al., 2015; Tili et al., 2017) and neurodevelopmental disorders (Ha and Kim, 2014).

Among miRNAs, microRNA-31 (MIR31) is increased in colorectal cancer (Wang et al., 2009) and patients with IBD (Béres et al., 2017). MIR31 is also proven to be up-regulated during IBD-associated neoplastic transformation (Olaru et al., 2011). Targeting MIR31 pathways involved in the inflammatory response paves the way for disease treatments (Yu et al., 2021). MIR31 can promote epithelial regeneration during skin wound healing by mediating inflammatory signaling (Shi et al., 2018), and target IL-25 to regulate IL-12/23-mediated Th1/Th17 inflammatory responses during colitis (Shi et al., 2016). We previously showed that MIR31 promotes the self-renewal of mammary stem cells and mammary tumor growth by regulating the WNT signaling pathway (Lv et al., 2017). Our recent study also indicated that MIR31 can reduce inflammatory responses in colonic epithelium by inhibiting inflammatory cytokines receptors, and promote epithelial regeneration through regulating WNT and Hippo signaling pathways (Tian et al., 2019). However, the mechanism underlying the regulation of MIR31 in these pathways is not yet clear. More importantly, the questions of whether and how these two functions (i.e., inflammatory response inhibition and epithelial regeneration promotion) compete for MIR31 during colitis require further clarification.

Experiment-based network modeling is a powerful approach to investigate the biological dynamics in animals (Li et al., 2021b, 2022), plants (Wu et al., 2021), and bacteria (Liu et al., 2021), and is also widely applied to investigate the role of miRNAs (Lai et al., 2018). To systematically analyze the regulatory mechanism of MIR31 in inflammation and epithelial regeneration, experimental analysis is performed and a corresponding phenomenological model is proposed. Experimental observations suggest that the expression of MIR31, phosphorylated p65 (p-65), and phosphorylated STAT3 (p-STAT3) present an “adaptation” behavior in dextran sulfate sodium (DSS)-induced mouse colitis (Ma et al., 2009), which are well reproduced by our model. Dynamics of the number of proliferative epithelial cells and the expression of p-65 and p-STAT3 in MIR31 knockout (KO) mice are also predicted and validated, indicating that MIR31 restrains the activation of p65 and STAT3 but promotes epithelial regeneration. Further analysis suggests the behavior of the epithelial cell number exhibits the “spring-like effect.” Acting as an external force, DSS drives the system to a “spring compressing process” by reducing the epithelial cell number which is analogous to spring length. MIR31 acts as the intrinsic force and fine-tunes the epithelial cell number, propelling the system to a “spring compression state” with a small cell number and a high level of MIR31. Highly expressed MIR31 then accelerates epithelial regeneration by promoting cell proliferation after the withdrawal of DSS, corresponding to “spring recovery process.” Overall, this study provides quantitative new insights into the regulatory mechanism of MIR31, offering possible therapeutic strategies for colitis and related diseases.

## Materials and methods

### Animal experiments

Wild-type (WT) C57BL/6 mice were purchased from Beijing Vital River Laboratory Animal Technology Company (Beijing). MIR31 knockout (MIR31-KO) and control mice have been previously described (Tian Y. et al., 2017). About three to four 8-week-old mice were used at each time points for analysis in this study. All mice were fed under specific pathogen-free conditions. All experiments were approved by the guidelines of the Institutional Animal Care and Use Committee of China Agricultural University.

### DSS treatment

Adult mice were fed 3.5% wt/vol DSS with molecular weight 36,000 to 50,000 (MP Biochemicals, Santa Ana, CA) in drinking water for 5 days, and then DSS was withdrawn for recovery for 3 days. Tissues were harvested at the indicated time points. The colonic tissues were fixed in 4% paraformaldehyde for 24 h, embedded in paraffin, sectioned, and stained with hematoxylin and eosin.

### Immunofluorescence staining

The paraffin-embedded 5 μm sections were dehydrated with graded alcohol, and antigen retrieval was performed by heating slides for 20 min in 0.01 M citrate buffer (pH 6.0) with a microwave oven. The sections were blocked for 1 h at room temperature with blocking buffer (Beyotime) and incubated with primary antibodies at 4°C overnight. Next, the sections were washed with PBS three times, each time for 5 min, incubated with secondary antibodies for 1 h at room temperature and counterstained with DAPI. For immunohistochemistry staining, antigen retrieval was performed by heating slides for 20 min in 0.01 M citrate buffer (pH 6.0) with a microwave oven. Then, the sections were stained according to the SP Kit (ZSGB-Bio) manufacturer’s instructions. The following primary antibodies were used: Ki67 (1:1,000, ab15580, Abcam), β-catenin (1:500, sc-7,963, Santa Cruz), pStat3 (1:400, 9,145, CST), and p65 (1:1,000, 8,242, CST). The following secondary antibodies were used: Alexa Fluor 488 goat anti-mouse IgG (H + L) and Alexa Fluor 594 goat anti-rabbit IgG (H + L).

### *In situ* hybridization

The MIR31 *in situ* hybridization assay was performed as described previously with modifications (Tian Y. et al., 2017). Digoxigenin-labeled LNA probes (Exiqon, Vedbaek, Denmark) were used following the manufacturer’s protocol. Both digoxigenin-labeled MIR31 and scrambled LNA probes (Exiqon) were hybridized at 61°C. The U6 probe was used as a positive control. *In situ* signals were detected by staining with anti-digoxigenin-AP antibody (Roche, Basel, Switzerland) and developed using BM purple substrate (Roche).

### Model construction

In WT model, the DSS-induced inflammatory response is composed of four processes: DSS-induced inflammatory cytokines production, MIR31 induction, MIR31-inhibited inflammatory cytokines production and MIR31-promoted epithelial regeneration. While in KO model, MIR31 is knocked out and the correlated processes of MIR31 exist no more. We only consider the epithelial regeneration by intrinsic cell proliferation. A system of ordinary differential equations (ODEs) is a common approach to describe dynamics of biochemical reactions and interactions of signaling molecules (Li et al., 2021a). Based on the Hill equation, the evolution of molecular concentrations with time in the model can be described


$$\frac{dY_{i}}{dt}=\overset{s}{\underset{j}{\sum }}k_{ij}\times \frac{Y_{j}{}^{n}}{l_{ij}^{n}+Y_{j}{}^{n}},i=1,\ldots m$$

where d*Yi/dt* is the rate at which the concentration of molecule i changes over time. *m* represents the number of molecules with concentration *dYi*. *s* denotes the number of reactions with rate *k_ij_*, the half-saturation constant *l_ij_* and the Hill coefficient n. *Yj* is the concentration of molecule involved in the reaction. The ODEs that describe the reactions of different modules in the signaling model are shown in Supplementary Text.

### Parameter values and initial amount selection

All parameters in the signaling model are first limited to the typical biological ranges depending on the types of reaction (Alon, 2006) and further estimated based on the experimental results (Tian et al., 2019). The initial values of the parameters are random selected to avoid convergence to local minimums, and then mainly determined by a global optimization method to minimize the deviations between simulation results and experimental results, including the expressions of MIR31, p-p65 and p-STAT3, as well as the number of proliferative cells. The descriptions, values and units of all parameters in the signaling model are given in Supplementary Tables S1, S2.

## Results

### “Adaptation” behavior of MIR31 In DSS-induced colitis

**Figure 1:**
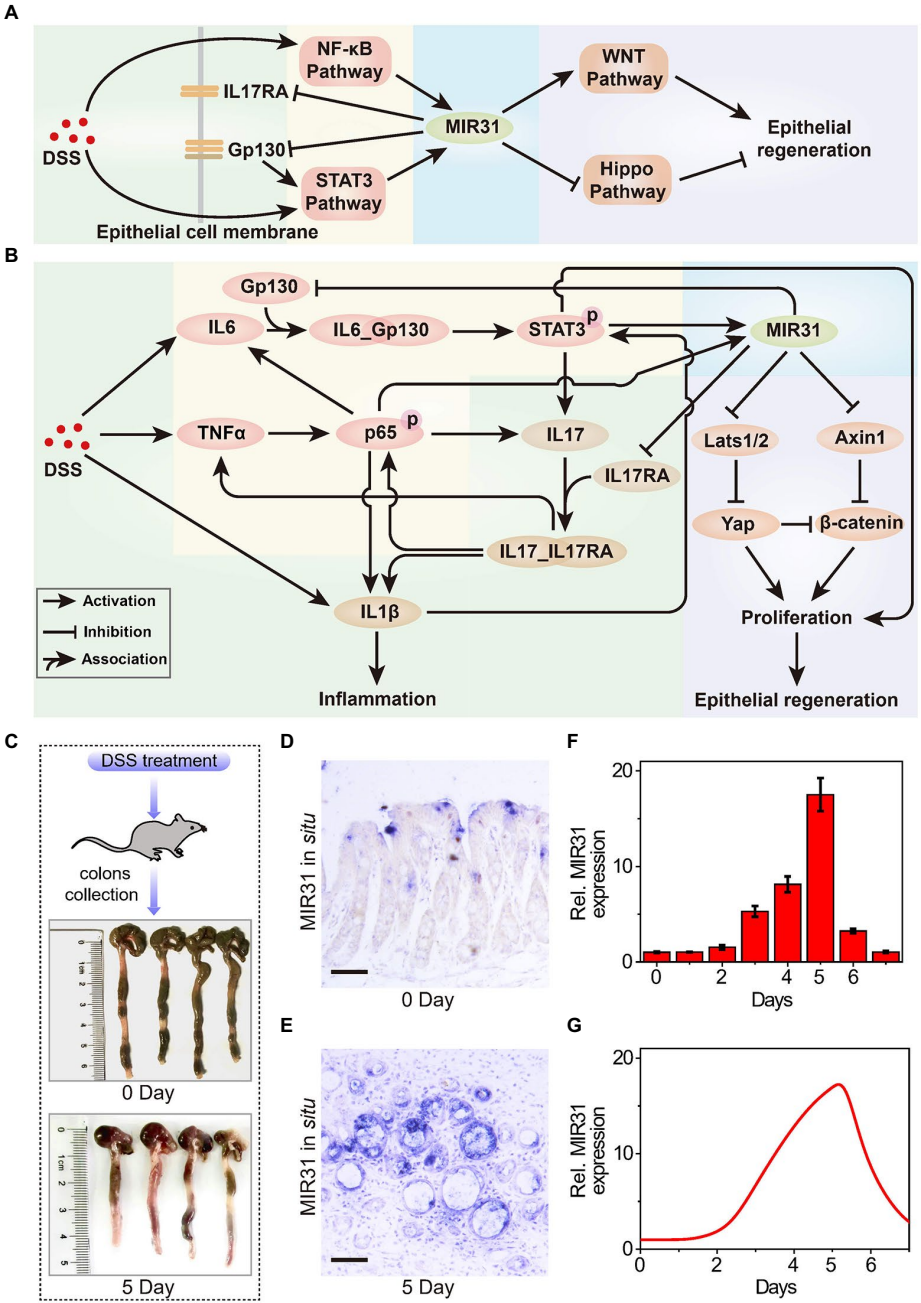
The role of MIR31 in DSS-induced colitis signaling network. **(A)** Simplified signal transduction network of DSS-induced colitis to show the main interactions between MIR31 and four pathways of NF-κB, STAT3, WNT and Hippo signals. **(B)** Detailed signal transduction network. The network is composed of four modules, which are highlighted by different backgrounds. The green background is for the module of DSS-induced inflammatory cytokine production, the yellow for the module of MIR31 induction, the blue for the module of MIR31-inhibited inflammatory cytokine production, and the purple for the module of MIR31-promoted epithelial regeneration. **(C)** WT mice used in our experiments and colonic tissues collected from WT mice at 0 day and 5 day of DSS treatment. **(D)** *In situ* hybridization for MIR31 in normal colons without DSS treatment. **(E)** *In situ* hybridization for MIR31 in colons from mice treated with DSS for 5 days. **(F)** qRT–PCR analysis showing MIR31 expression levels in the colonic epithelium from DSS-treated mice at the indicated time points. *n* = 4 at each time point. **(G)** Simulation results of the MIR31 expression level over time in DSS-induced WT model.

A feasible theory of the pathogenesis of IBD is that the barrier of intestinal epithelial cells loses its function, with luminal organisms or their products entering the lamina propria. One of the most widely used IBD animal models is the DSS-induced colitis mice model (containing a simple microbiota), which is similar to UC in terms of pathology, pathogenesis and other aspects (Wirtz and Neurath, 2007; Solomon et al., 2010; Mizoguchi, 2012; Nguyen et al., 2015; Eichele and Kharbanda, 2017). A common mechanism for DSS-induced colitis involves damage to the intestinal epithelial barrier, which allows luminal bacteria and associated antigens to enter the mucosa (Figure 1A). The entry induces immune responses from immune cells (e.g., macrophages and T cells) in the epithelial lamina propria and the release of inflammatory cytokines, triggering acute inflammation (Kiesler et al., 2015). Receptors (Gp130 and IL17RA) of inflammatory cytokines localize to the colonic epithelium (Zhang et al., 2006; Ernst et al., 2014) and activate epithelial STAT3 and NF-κB signaling pathways (De Robertis et al., 2011), inducing MIR31 activation. In addition, MIR31 promotes epithelial regeneration by activating the WNT signaling pathway and inhibiting the Hippo signaling pathway through several target genes, such as Axin1 and Lats1/2 (Tian et al., 2019).

DSS-induced inflammatory response is a complicated biological process that involves different cells, such as macrophages and intestinal epithelial cells (IECs; Saleh and Trinchieri, 2011), and the major biological network is shown in Figure 1B. The signaling network is composed of four modules, including the production of DSS-induced inflammatory cytokine (green background), the induction of MIR31 (yellow background), the inhibition of inflammatory cytokine production by MIR31 (blue background), and the epithelial regeneration promoted by MIR31 (purple background). After DSS administration, immune cells can recognize the agents and rapidly release inflammatory cytokines IL1β and IL6 (Francescone et al., 2015), accompanied by increased TNFα expression (Zelov and Hošek, 2013; Kany et al., 2019). When TNFα reaches high concentrations, the NF-κB signaling pathway is activated in the form of phosphorylated p65 (p-p65) *via* the canonical pathway (Giridharan and Srinivasan, 2018), which promotes the production of IL1β (Kelley et al., 2019) and further exacerbates inflammation. The NF-κB signaling pathway also induces IL6 expression, which can activate the STAT3 signaling pathway (Ernst et al., 2014). Then, the activated STAT3 (p-STAT3) promotes the secretion of the inflammatory cytokine IL17, in turn facilitating the activation of the NF-κB pathway (Razi et al., 2019). In addition, IL1β can slightly accelerate the activation of STAT3 (Parker et al., 2015; van de Wetering et al., 2020). We previously identified one STAT3 and two NF-kB binding sites in the promoter of MIR31 (Lv et al., 2017; Tian Y. et al., 2017) and proved that the induction of MIR31 is due to the activation of NF-κB and STAT3 signaling pathways (Tian et al., 2019). Moreover, our former study also demonstrated that MIR31 directly inhibits inflammation through the suppression of receptor Gp130 and receptor IL17RA (Tian et al., 2019). The canonical WNT signaling pathway is a key regulator of epithelial regeneration (Moparthi and Koch, 2019). Hippo signaling pathway also drives epithelial regeneration in colon after DSS-induced injury (Deng et al., 2018; Xie et al., 2021). We previously revealed that MIR31 promotes epithelia regeneration by modulating the WNT and Hippo pathways, restoring the ability of epithelial cells to resist inflammation (Tian et al., 2019). Besides, Axin1 and β-catenin, as well as Lats1/2 and Yap, are the two groups of important transducers in WNT and Hippo pathways, respectively.

To qualitatively investigate the regulatory mechanism of MIR31 in colitis, experimental analysis is performed to explore the dynamics of the core transducers, i.e., MIR31, p-p65, and p-STAT3, in response to the DSS-induced colitis in WT mice. Figure 1C shows the normal colonic tissues (0 days) and colonic tissues at 5 days of DSS treatment from WT mice. *In situ* hybridization for MIR31 in colons in Figures 1D,E is obtained from untreated WT mice and WT mice after 5 days of DSS treatment, respectively. As the quantified experimental data shows, an obvious up-regulation of MIR31 in the colonic epithelium is observed (Figure 1F). After 5-day DSS administration, MIR31 expression increased to a high peak and then rapidly decreased to the baseline of pretreatment after DSS withdrawal, presenting an “adaptation” behavior (Figure 1F). A phenomenological network model is also developed based on the signaling pathways shown in Figure 1B, which can provide a more quantitative diagram to further explore the role of the signaling pathways in the pathogenesis of colitis. The model is described by a cast of ordinary differential equations and the complete model descriptions are presented in the Supplementary material. Simulation results suggest that our model can well reproduce the “adaptation” behavior of MIR31 expression after DSS treatment (Figure 1G).

**Figure 2:**
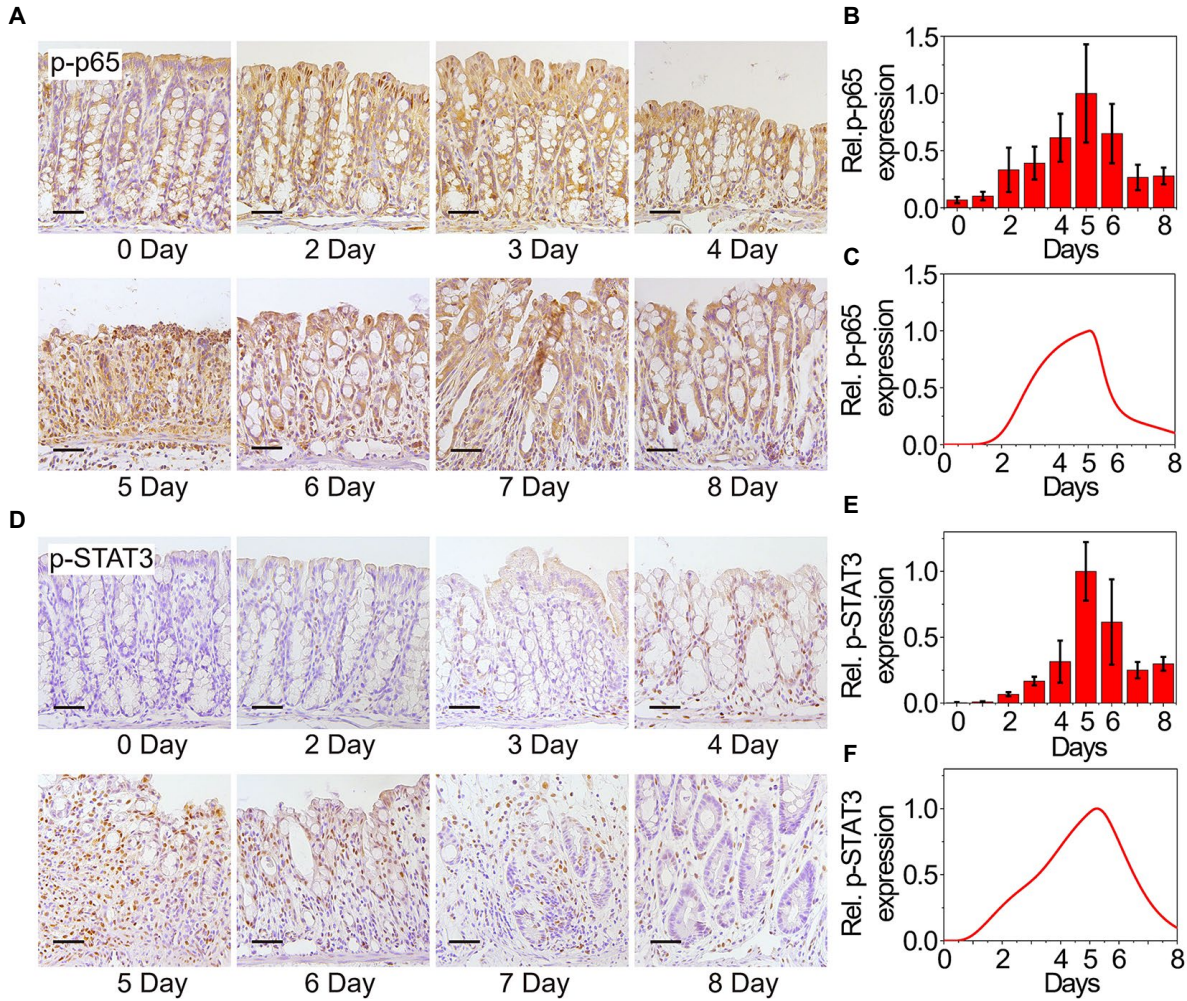
Experimental and modeling results of p65 and STAT3 activation. **(A)** Immunohistochemistry for p65 in the colons from WT mice at the indicated time points following DSS treatment. n = 3 at each time point. **(B)** Statistical histogram of experimental immunohistochemistry results for p65 in panel **(A)**. **(C)** Simulation results of p-p65 expression in WT model over time after DSS treatment. P65 is phosphorylated when it is transferred to the nucleus. **(D)** Immunohistochemistry for p-STAT3 in the colons from WT mice at the indicated time points following DSS treatment. *n* = 3 at each time point. **(E)** Statistical histogram of experimental immunohistochemistry results in panel **(D)**. **(F)** Simulation results of p-STAT3 expression in WT model over time after DSS treatment. Here, the results in **(B,C,E,F)** are normalized to the corresponding maximum of p-p65 and p-STAT3, respectively.

Dynamical expressions of the two core transducers p-p65 and p-STAT3 that directly facilitate the activation of MIR31 are quantified as well. Abnormal increases in p-p65 are associated with many chronic diseases such as rheumatoid arthritis and IBD (Simmonds and Foxwell, 2008; Giridharan and Srinivasan, 2018). With DSS treatment, the NF-κB signaling pathway is activated, accompanied by a sustained increase in p-p65, which can be detected in the nucleus by immunohistochemistry (Figure 2A). Then, p-p65 expression is gradually restored to its initial level along with the reduction of inflammation. The quantified experimental data shown in Figure 2B indicates that p-p65 also presents an “adaptation” behavior, which can be reproduced by our model (Figure 2C). The p-STAT3 mediated inflammation is shown by immunohistochemistry in Figure 2D and the experimental result suggests the “adaptation” behavior of p-STAT3 (Figure 2E). Simulation results of p-STAT3 and p-p65 expressions suggest that the “adaptation” behavior of p-STAT3 and p-p65 are slightly different. After 5 days of DSS treatment, p-STAT3 seems to decrease at a constant rate (Figure 2F), while p-p65 declines at first rapidly and then gradually (Figure 2C). When quantifying the experimental results, ilastik, an interactive machine learning for (bio)image analysis, was used to binarily classify the images firstly (Berg et al., 2019). Then the objects in the processed images were statistically analyzed with ImageJ which was widely used in the biological sciences and other projects (Schindelin et al., 2012).

### MIR31 suppresses p-p65 and p-STAT3, but accelerates the recovery of epithelia in colitis

**Figure 3:**
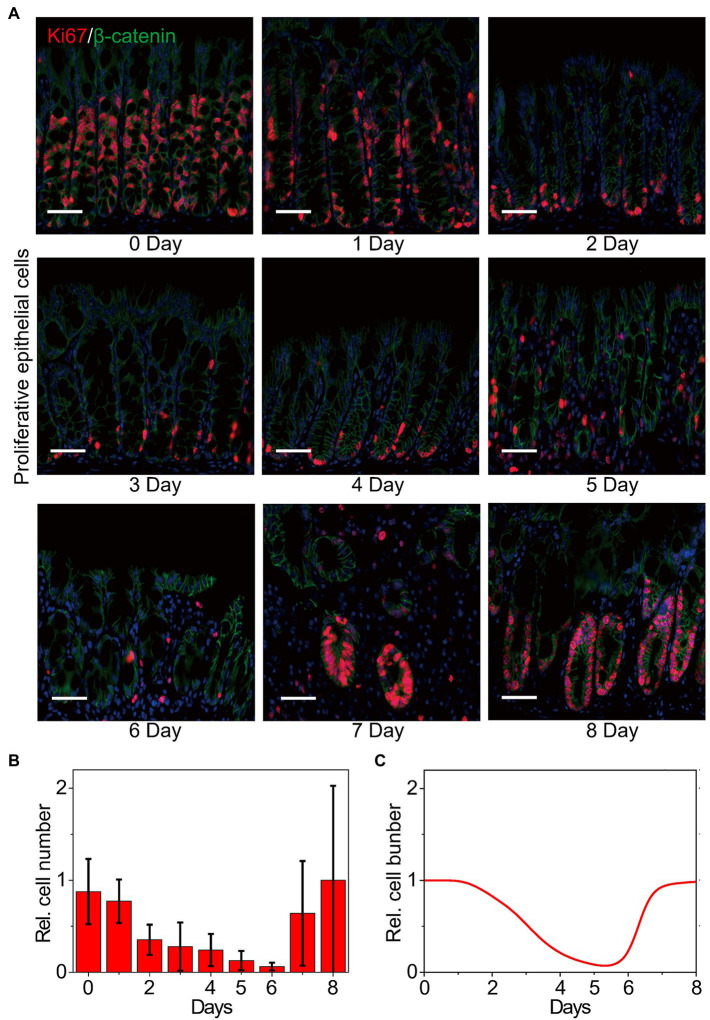
Dynamics of the number of proliferative epithelial cells. **(A)** Double immunofluorescence for Ki67 and β-catenin in colons from WT littermates at the indicated time points following DSS treatment. *n* = 3 at each time point. **(B)** Statistical histogram of experimental results for the proliferative cell number in panel **(A)**. **(C)** Simulation results of the proliferative cell number in WT model over time after DSS treatment. Here, the cell numbers of proliferative epithelial cells are normalized by the maximum value.

Dysregulation of the intestinal epithelium homeostasis has been detected in IBD (Podolsky, 2002). The intestinal epithelium homeostasis depends on the IECs, the intestinal microbiota, and the intestinal immune system (Hooper and Macpherson, 2010), in which the IECs provide a mucosal barrier to segregate host immune system and commensal bacteria (Roda et al., 2010; Peterson and Artis, 2014; Okumura and Takeda, 2017). The proliferation and apoptosis of IECs are also studied to evaluate the clinical symptoms in colitis (Renes et al., 2002). Thus, the number change of proliferative cells is considered to characterize the inflammatory process and the recovery of epithelia in colitis. Figure 3A shows the double immunofluorescence of the proliferative cells at different time points in WT mice upon DSS treatment. For double immunofluorescence of Ki67 and β-catenin in the colons, we count the number of proliferative cells per crypt. The quantified data indicates that the number of proliferative cells declines first and then recovers to the initial state (Figure 3B). Dynamics of the proliferative cells can also be well reproduced by our model (Figure 3C), confirming that our model has the potential for exploring the signaling properties and giving mechanistic insights of MIR31 in colitis.

**Figure 4:**
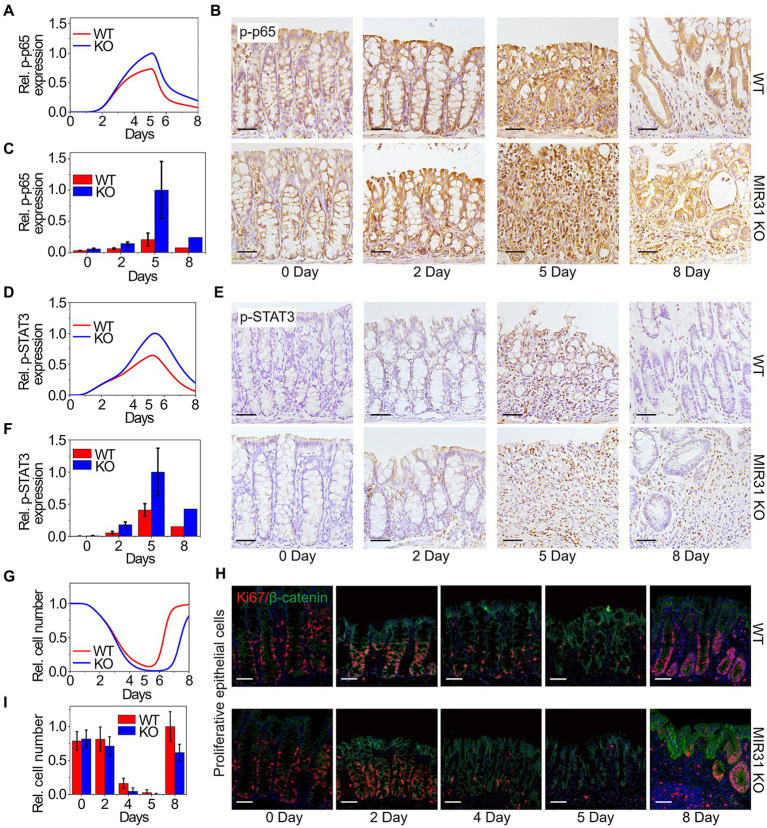
Modeling predictions and experimental confirmations in WT and MIR31-KO mice. **(A,D,G)** Model predictions of p65 activation **(A)**, STAT3 activation **(D)** and the proliferative cell number **(G)** for the WT and MIR31-KO models over time after DSS treatment. **(B,E,H)** Immunofluorescence for p65 **(B)**, p-STAT3 **(E)**, and Ki67/β-catenin **(H)** in colons from WT and MIR31-KO mice at the indicated time points following DSS treatment. *n* = 3 at each time point. **(C,F,I)** Corresponding statistical histograms of experimental results for p65 in panel **(B)**, for p-STAT3 in panel **(E)**, and for the proliferative cell number in panel **(H)**, respectively. All the results in **(A,C,D,F,G,I)** are normalized to the corresponding maximum values.

We next apply our model to quantitatively dissect the functional roles of MIR31 in colitis. Simulation results predict that deletion of MIR31 in mice increases the peak value of p-p65 compared to the WT mice (Figure 4A). Immunofluorescence experimental analysis for p-p65 is performed in MIR31-KO mice to validate the prediction (Figure 4B). As the quantified results shown (Figure 4C), deletion of MIR31 indeed amplifies the “adaptation” behavior of p-p65, revealing that MIR31 suppresses the activation of p65 in colitis. Similar predictions and experimental validations of p-STAT3 suppressed by MIR31 are also determined (Figures 4D,F). Thus, both the activation of p65 and STAT3 are restrained by MIR31 in colitis. The role of MIR31 in mediating the proliferative cell number is investigated as well. As shown in Figure 4G, our model predicts that the proliferative cell number decreases faster and recovers slower in the absence of MIR31. Analysis of immunofluorescence for Ki67 and β-catenin in MIR31-KO and WT mice is performed (Figure 4H) and our prediction matches well with the quantified experimental results (Figure 4I). Hence, above observations determine that MIR31 regulates inflammatory response through suppressing p65 and STAT3 activation, but promoting the recovery of epithelia during colitis.

### MIR31-involved reactions in determining proliferative epithelial cell number

**Figure 5:**
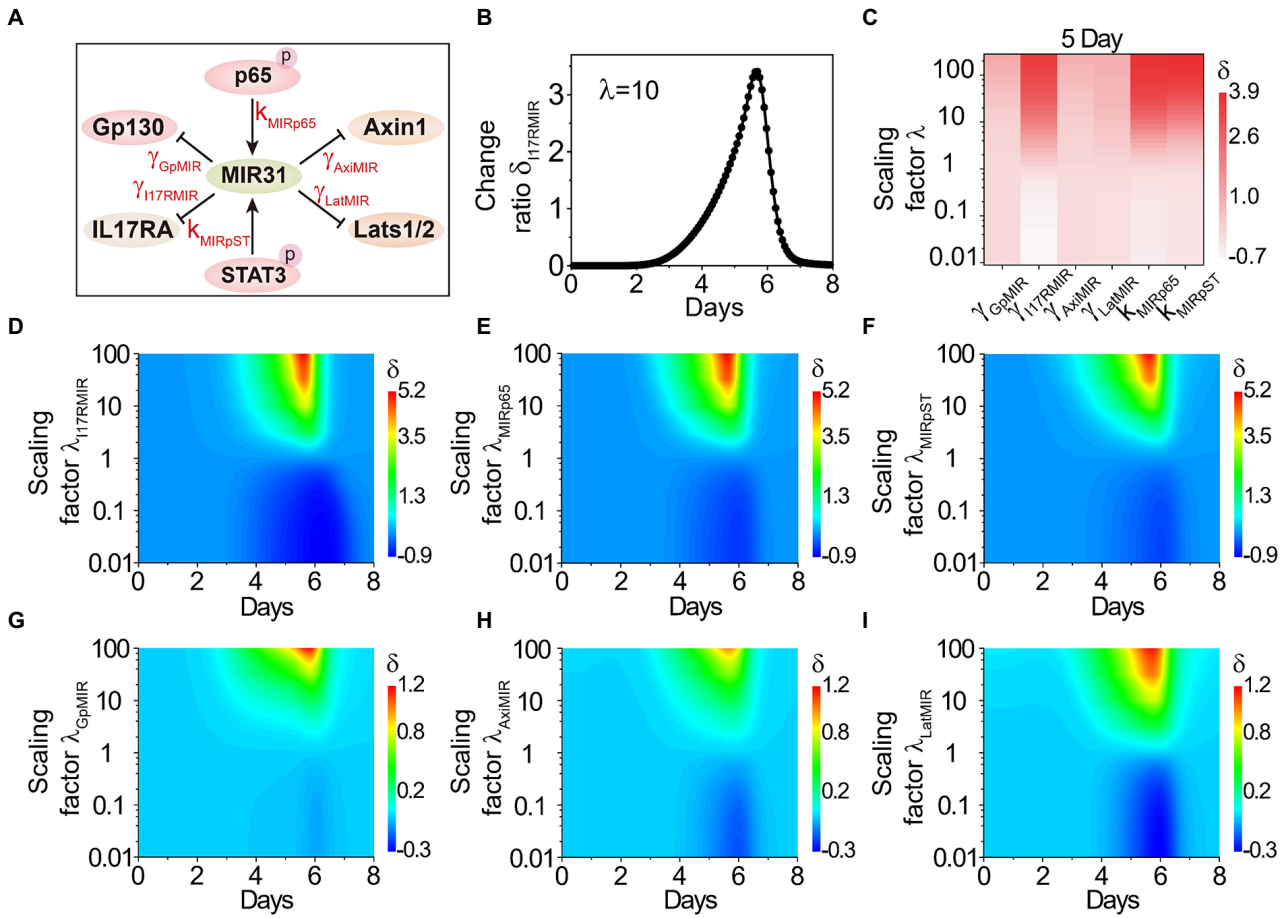
The effects of MIR31-involved reactions on the proliferative cell number. **(A)** The relationship between MIR31 and the related six proteins Gp130, IL17RA, Axin1, Lats1/2, p-p65 and p-STAT3. **(B)** The change ratio *δ* of the proliferative cell number varies with the scaling factor *λ* = 10 for the inhibition of MIR31 on IL17RA (i.e., γ*_I17RMIR_* increases with 10 times). **(C)** The variations *δ_i_****(t)*** for the inhibitions of MIR31 on Gp130, IL17RA, Axin1, and Lats1/2 (γ*_GpMIR_*, γ*_I17RMIR_*, γ*_AxiMIR_*, γ*_LatMIR_*), and the promotions of p-p65 and p-STAT3(*k_MIRp65_* and *k_MIRpST_*) on MIR31 with the scaling factor *λ* at 5 days of DSS treatment. **(D**–**I)** The change ratio *δ_i_*(*t*) as a function of time under continuous changes in the scaling factors *λ_i_* for all six parameters.

To dissect whether and how MIR31 mediate or is mediated by the transducers in colitis, we further analyze the effect of MIR31-involved reactions on the proliferative epithelial cell number, including the inhibitions of MIR31 on Gp130, IL17RA, Axin1 and Lats1/2 (represented by parameters γ*_GpMIR_*, γ*_I17RMIR_*, γ*_AxiMIR_* and γ*_LatMIR_*, respectively), and the promotions of p-p65 and p-STAT3 on MIR31 (represented by *k_MIRp65_* and *k_MIRpST_*, respectively) as shown in Figure 5A. We scale the parameters by multiplying the factor of *λ_i_* and define the change ratio *δ_i_(t)* of the proliferative cell number as *δ_i_(t) = |N_λi_(t)-N(t)|/N(t)*. *N_λi_(t)* is the proliferative cell number in the modified model with the scaling factor of *λ_i_*, *i* represents the corresponding reaction parameters, and *N(t)* is the proliferative cell number in the WT model.

We first discuss the effect of γ*_I17RMIR_* (the inhibition strength of MIR31 on IL17RA) on the proliferative cell number. As shown in Figure 5B, when the inhibition strength γ*_I17RMIR_* is increased by 10 times, the proliferative cell number increases significantly, indicating that the inhibition of MIR31 on IL17RA exhibits a strong impact on the inflammatory process. Effects of all the six MIR31-involved reactions on the proliferative cell number are discussed. The corresponding strengths change from 0.01 fold to 100 fold by tuning the scaling factor *λ_i_* from 0.01 to 100, and the variations of *δ_i_(t)* at 5 days of DSS treatment are shown in Figure 5C. One can see that the reaction of MIR31 inhibiting IL17RA (γ*_I17RMIR_*), and the reactions of p-p65 (*k_MIRp65_*) and p-STAT3 (*k_MIRpST_*) promoting MIR31 exhibit significant effects on the changes of proliferative epithelial cell number. The impacts of enhanced strengths on the proliferative cell number are greater than those of decreased strengths. While the variation of the other reactions, i.e., the inhibitions of MIR31 on Gp130, Axin1, and Lats1/2 (γ*_GpMIR_*, γ*_AxiMIR_* and γ*_LatMIR_*), barely influence the proliferative cell number (Figure 5C). Dynamic evolutions in the change ratio of the proliferative cell number *δ_i_(t)* as a function of time when the scaling factor *λ_i_* is varied continuously are studied and shown in Figures 5D–I, which further indicate that the parameters such as γ*_I17RMIR_*, *k_MIRp65_* and *k_MIRpST_* are still important on time scales, while the parameters of γ*_GpMIR_*, γ*_AxiMIR_* and γ*_LatMIR_* still exhibit little impacts. These results further display the significant roles of the reaction of MIR31 inhibiting IL17RA (Figure 5D), and the reactions of p-p65 (Figure 5E) and p-STAT3 (Figure 5F) promotion on MIR31 on the proliferative epithelial cells.

### Competition of MIR31 for inflammation inhibition and regeneration promotion

**Figure 6:**
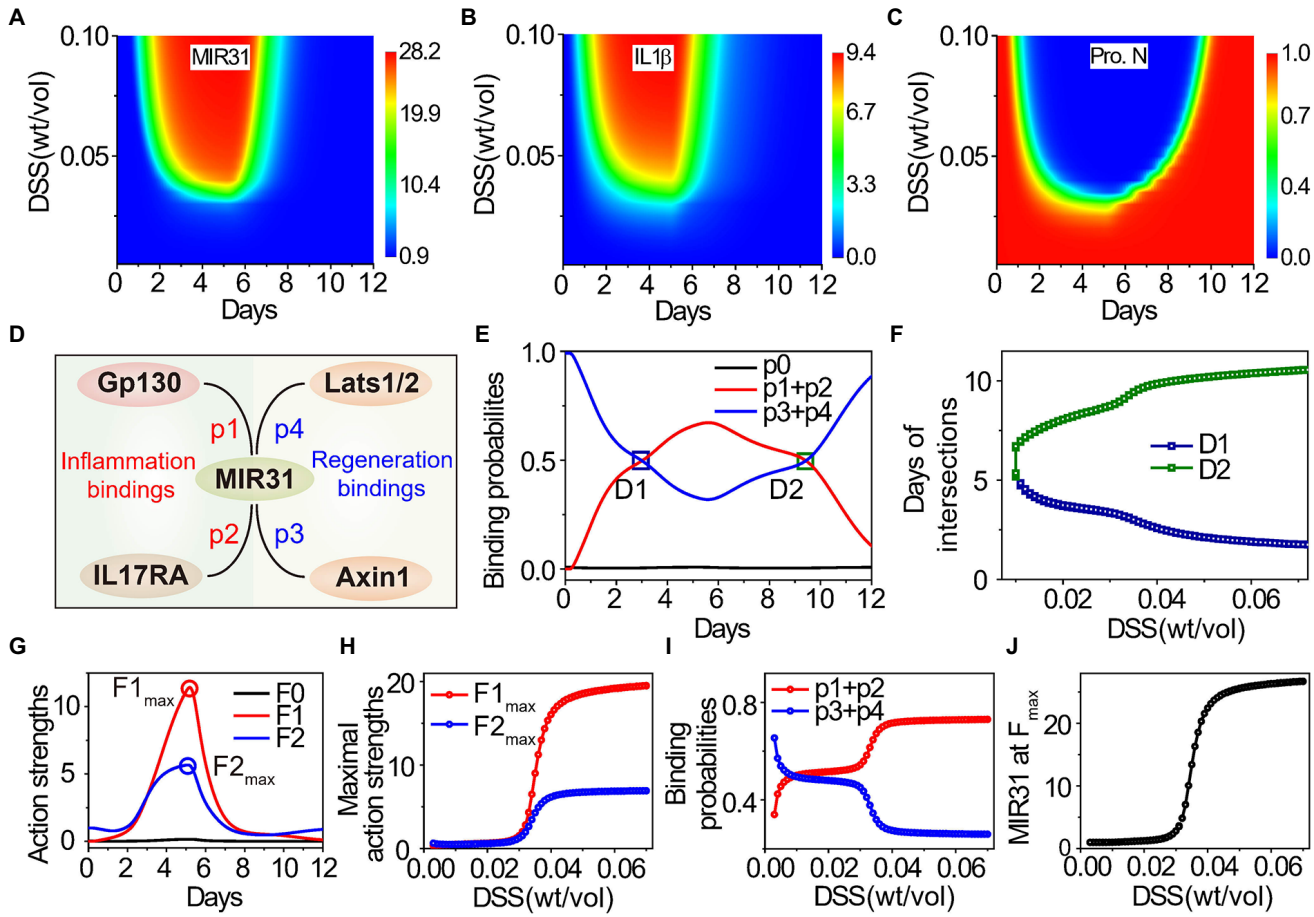
Effects of DSS concentration on inflammatory response and epithelial regeneration. **(A**–**C)** Dynamic results of MIR31 expression **(A)**, IL1β expression **(B)**, and the proliferative cell number **(C)** under continuous changes in DSS concentration. **(D)** The probabilities of MIR31 binding to Gp130 (*p1*), IL17RA (*p2*), Axin1 (*p3*), and Lats1/2 (*p4*). **(E)** Dynamics of the probability assignment of MIR31, where *p1* + *p2* and *p3* + *p4* correspond to MIR31-inhibited inflammation probability and MIR31-promoted regeneration probability, respectively, and *p0* represents the probability that MIR31 is in a resting state. **(F)** The influence of DSS concentration on two intersections D1 and D2 of the two probability curves in **(E)**. **(G)** Dynamics of the action strengths of MIR31-inhibited inflammation and MIR31-promoted regeneration. The influence of DSS concentration on the maximal action strengths **(H)** and on the probabilities of MIR31-inhibited inflammation and MIR31-promoted regeneration **(I)** and the MIR31 expression **(J)** corresponding to the two maximal action strengths in **(G)**. The results of **(C)** are compared with the maximum proliferative cell number. In **(E,G)**, MIR31 is divided into three categories: MIR31 in the resting state (black), MIR31 inhibiting inflammation (red) and MIR31 promoting regeneration (blue).

Previous studies indicate that the DSS-induced inflammation response is concentration dependent (Naito et al., 2003; Perše and Cerar, 2012), while the underlying regulatory mechanism remains unclear. To address this issue, quantitative analysis of the influence of DSS concentration in colitis is performed. Figures 6A–C show the dynamics of MIR31, IL1β, and the proliferative cell number, under continuous variation in DSS concentrations. Low concentrations (<2.5% wt/vol) of DSS treatment barely affect the signaling dynamics (Figures 6A–C). With high DSS concentrations, MIR31 is increased rapidly (Figure 6A), leading to the secretion of the inflammatory cytokine IL1β and inflammation induction during the first 5 days (Figure 6B). The induction of inflammation results in a significant decrease of the proliferative cell number (Figure 6C).

Since MIR31 can both suppress inflammation and promote epithelial regeneration (Figure 4), the competition of the two functional roles for MIR31 as well as the influence of DSS concentration on such competition are subsequently discussed. There are four major MIR31 target proteins, Gp130, IL17RA, Axin1, and Lats1/2, with certain probabilities of acting on inflammation and regeneration in colitis (Figure 1B). The probability of MIR31 binding to Gp130 and IL17RA are defined as *p1* and *p2*, which mainly contribute to inflammatory responses (Figure 6D). The probability of binding to Axin1 and Lats1/2 are defined as *p3* and *p4*, which mediates the epithelial regeneration. The constraint is *p1(t)* + *p2(t)* + *p3(t)* + *p4(t)* = 1-*p0(t)*, where *p0* represents the probability of MIR31 in a resting state without any binding. MIR31 inhibits inflammation with a probability of *p1* + *p2* and promotes regeneration with a probability of *p3* + *p4*. Dynamics of the MIR31 binding probability assignment for inhibiting inflammation and promoting regeneration are plotted in Figure 6E.

In healthy conditions without DSS (0 days), the main function of MIR31 is to promote regeneration. With DSS administration, the binding probability of MIR31-inhibited inflammation gradually increases (Figure 6E, red line) and the binding probability of MIR31-promoted regeneration conversely decreases (Figure 6E, blue line), indicating that the major function of MIR31 changes from regeneration promotion to inflammation inhibition. After DSS is withdrawn, the binding probability of MIR31-inhibited inflammation decreases, and correspondingly, the binding probability of MIR31-promoted regeneration increases. As shown in Figure 6E, the two binding probabilities intersect at the 3rd day and 10th day with intersections D1 and D2. This indicates a larger binding probability of MIR31 for inflammation inhibition than for regeneration promotion during the 3rd to the 10th days to prevent the inflammation caused by DSS.

To discuss the influence of DSS concentration on the competition of MIR31-inhibited inflammation and MIR31-promoted regeneration, the time corresponding to D1 and D2 with the change in DSS concentration is studied. As the result shows in Figure 6F, when the DSS concentration is smaller than 1.1% wt/vol, the binding probability of MIR31-promoted regeneration is typically larger than the binding probability of MIR31-inhibited inflammation without intersection, suggesting that the major function of MIR31 is to promote regeneration in weak DSS-induced colitis. The probability of MIR31-inhibited inflammation increases gradually as the DSS concentration increases. In the case of strong DSS-induced colitis (DSS > 3.0% wt/vol), the major function of MIR31 changes to inhibiting inflammation rather than to promoting regeneration (Figure 6F).

MIR31 expression ([MIR31]) increases obviously after DSS treatment (Figure 1F). Thus, the action strength of MIR31-inhibited inflammation can be defined as *F1(t) = [MIR31(t)] × (p1(t) + p2(t))*, and the action strength of MIR31-promoted regeneration as *F2(t) = [MIR31(t)] × (p3(t) + p4(t))*, giving the total action of MIR31 on the system as *F(t) = F0(t) + F1(t) + F2(t)*, where *F0(t) = [MIR31(t)] × p0(t)* represents the action strength of MIR31 in a resting state without any binding. Interestingly, the action strength of MIR31-inhibited inflammation (*F1*) increases significantly after DSS treatment, while the action strength of MIR31-promoted regeneration (*F2*) shows less enhancement in Figure 6G. The maximal action strength is obtained at approximately the 5th day.

Considering the influence of DSS concentration on the action strengths of MIR31, as shown in Figure 6H, the maximal action strengths of MIR31 remain at low levels when the DSS concentration is small, while they increase rapidly with increasing DSS concentration when DSS is larger than 3.0% wt/vol, especially for the maximal action strength of MIR31-inhibited inflammation (*F1_max_*). Surprisingly, we found that the probability of MIR31-promoted regeneration decreases in a stepwise manner with increasing DSS concentration (Figure 6I, blue line), which is contrary to the trend of the action strength of MIR31-promoted regeneration (Figure 6H, blue line). Further analysis determines the reason for this to be the corresponding increase in MIR31 expression as shown in Figure 6J being much greater than the decrease in the probability of MIR31-promoted regeneration (Figure 6I). Thus, the changes in the binding probabilities of MIR31 determine the transformation of its functions, while the expression level of MIR31 determines the action strengths of its functions for inhibiting inflammation and promoting regeneration.

## Discussion

Previous studies demonstrated that miRNAs are associated with various diseases including COVID-19 (Li et al., 2020; de Gonzalo-Calvo et al., 2021) and have the potential to be therapeutic targets (Guterres et al., 2020; Hum et al., 2021). MIR31 is identified as a key regulator in diseases (Valastyan and Weinberg, 2010; Liu et al., 2010a; Laurila and Kallioniemi, 2013; Stepicheva and Song, 2016). MIR31 acts as an oncogenic miRNA in lung cancer by targeting specific tumor suppressors for repression (Liu et al., 2010b). In addition, MIR31 is proven to be a target for inhibiting tumor growth and metastasis (Tian C. et al., 2017). The down-regulation of MIR31 disrupts cellular homeostasis and promotes the evolution and progression of prostate cancer (Lin et al., 2013b). The pathogenesis of colitis involves many complex signaling pathways that are related to various types of cells at tissue level (Perše and Cerar, 2012). Understanding the mechanism of MIR31 in colitis is therefore urgently needed for developing therapies for diseases.

Combining experimental analysis and a proposed phenomenological network model, we quantitatively explored the important roles of MIR31 in modulating inflammation and epithelial regeneration, and identified effective targets for clinical treatment of inflammation. Our study indicates that MIR31 exhibits an “adaptation” behavior in WT model of DSS-induced colitis and similar “adaptation” behavior also occurs in p-p65 and p-STAT3. The number of proliferative cells decreases gradually and then recovery to normal state after DSS treatment (Figure 3). In MIR31 KO model, the “adaptation” behavior of p-p65 and p-STAT3 is magnified, indicating the suppression of MIR31 on the activation of NF-κB and STAT3 signaling pathways. The number of proliferative cells decreases more quickly with fewer survival cells and recovers slower, suggesting a promotion of MIR31 on epithelial regeneration (Figure 4). As a novel therapeutic target, MIR31 has been extensively studied in various diseases such as colorectal cancer (Zhao et al., 2020), nasopharyngeal carcinoma (Wu et al., 2016). PEX5, a novel target of MIR31, is also proven to be a therapeutic option in hepatocellular carcinoma (Wen et al., 2020). Our analysis shows that the inhibition of MIR31 on IL17R, the promotions of p-p65 and p-STAT3 on MIR31 exhibit virtual influences on the number of proliferative cells, which can be considered as a potential therapeutic target in future studies.

**Figure 7:**
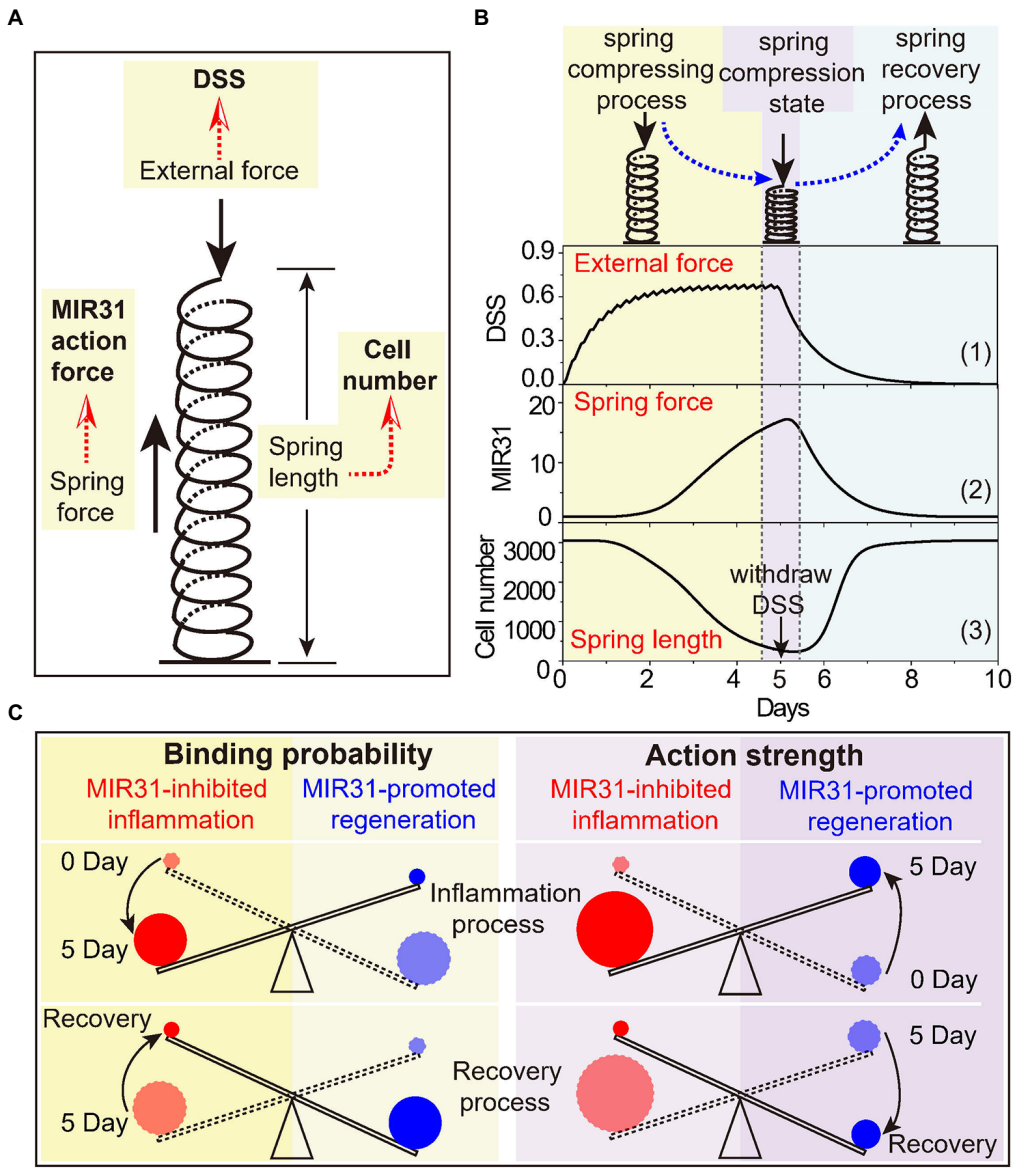
The spring-like effect and the seesaw model of MIR31 in balancing inflammatory and regenerative responses. **(A)** Schematic diagram of the spring-like effect of inflammatory response to DSS treatment. **(B)** The specific correspondence between the inflammatory response process to DSS and the spring system. Considering the trend of MIR31 expression trend was consistent with that of MIR31 action strength, it was used to characterize the trend of the MIR31 action strength. **(C)** The seesaw model of MIR31 competition mechanism in the WT model from the viewpoints of the binding probability (left) and the action strength of MIR31 (right).

To intuitively present the mechanism of MIR31, we propose that the MIR31 response process in colitis can be characterized by the “spring-like effect” (Figure 7A). In this analogy, DSS acts as the external pressure on the spring, the number of prolifertive cells is the spring length, and the MIR31 action strength, which involves MIR31-promoted regeneration and MIR31-inhibited inflammation, is the intrinsic spring force. With such a view, DSS drives the system into the “spring compressing process” and thus the cell number decreases. Meanwhile, MIR31 expression increases gradually to prevent DSS-induced colitis (Figure 7B). When DSS remains high, the action strength of MIR31 on inflammation also remains strong with a small cell number. Even immediately after DSS is withdrawn, the residual DSS in the system is still strong, which leads to low cell numbers. We name this state the “spring compression state.” Then, DSS becomes attenuated while the action strength of MIR31 on inflammation inhibition still holds dominant, resulting in a rapid increase in cell number. This process corresponds to the “spring recovery process,” in which MIR31 expression decreases, leading to a gradual reduction in the action strength of MIR31. Finally, the system returns to the normal state. Note that in the MIR31-KO model, the inflammatory response process can also be described by the “spring-like effect,” while the spring compressing process (compression state and recovery process) occurs on a time scale shorter (longer) than that in the WT model.

In our study, we simply compared the inflammatory response to “spring-like effect” as the response presents a process similar to that of a spring from compression to recovery. The inflammatory response is a nonlinear process that depends on time and external force. Actually, the process can also be regarded as a visco-elastic system, such as the “spring-dashpot model.” Recently, a visco-elastic system has been proposed in which the cytoplasm contributes to mitotic spindle positioning through its visco-elastic property (Xie et al., 2022). Besides, a commentary also defines this visco-elastic property as the “spring-like behavior” (Bai and Mitchison, 2022).

Due to the competitive binding mechanism for MIR31 to either inhibit inflammation or promote regeneration, as well as the influence of MIR31 level on the competition, we suggest that such a competition mechanism can be understood by a “seesaw model” (Figure 7C). The seesaw is balanced by MIR31-inhibited inflammation and MIR31-promoted regeneration. Based on the concept of the binding probabilities, the change in the seesaw is induced by the change of MIR31 binding probabilities to different proteins (Figure 6E). In detail, MIR31 displays an increased binding probability for inflammation inhibition and a decreased binding probability for regeneration promotion during the inflammation process (0 day–5 day). During the recovery process, there is a diminution in MIR31 binding probability for inflammation inhibition and an enhancement of MIR31 binding probability for regeneration promotion (5 day–10 day). Notably, a different mechanism can be obtained in the seesaw from the viewpoint of the MIR31 action strength. Both the action strengths of MIR31-promoted regeneration and MIR31-inhibited inflammation increase with time during the inflammation process, with the action strength of MIR31-inhibited inflammation gradually becoming dominant (0 day–5 day). However, during the recovery process, both the action strengths of MIR31-promoted regeneration and MIR31-inhibited inflammation decrease with time, with the action strength of MIR31-promoted regeneration finally becoming dominant (5 day–10 day).

In summary, the seesaw model and the spring-like effect for MIR31 functions highlight the importance of MIR31 in the inflammatory response process. MIR31 can effectively alleviate inflammation by inhibiting inflammatory cytokine receptors and can promote epithelial regeneration by modulating the WNT and Hippo signaling pathways. With the model, we suggest that the inhibition of MIR31 on cytokine receptors is crucial to inflammation control and can be regarded as a therapeutic target for drug design.

## Data availability statement

The original contributions presented in the study are included in the article/Supplementary material, further inquiries can be directed to the corresponding authors.

## Ethics statement

The animal study was reviewed and approved by the Institutional Animal Care and Use Committee of China Agricultural University.

## Author contributions

JQ developed the model and performed simulation. JQ and XL wrote the manuscript. CS and YT designed the experiments. CS implemented the experiments. JQ, CS, XL, and YY performed discussion of experimental data and modeling results. YY, YW, WL, and ZhiY helped for model discussion. XL, ZheY, and JS revised the paper, conceived the idea, and supervised the project. All authors contributed to the article and approved the submitted version.

## Funding

This work was supported by the National Natural Science Foundation of China (Grant Nos. 12090052, 11874310, 81772984, and 82025006), the National Science and Technology Major Project of the Ministry of Science and Technology of China (Grant Nos. 2021ZD0201900 and 2021ZD0201904), and the Fujian Province Foundation (Grant No. 2020Y4001).

## Conflict of interest

The authors declare that the research was conducted in the absence of any commercial or financial relationships that could be construed as a potential conflict of interest.

## Publisher’s note

All claims expressed in this article are solely those of the authors and do not necessarily represent those of their affiliated organizations, or those of the publisher, the editors and the reviewers. Any product that may be evaluated in this article, or claim that may be made by its manufacturer, is not guaranteed or endorsed by the publisher.
