# Supplementary material for: The spring-like effect of microRNA-31 in balancing inflammatory and regenerative responses in colitis
Source: Front Microbiol. 2022 Dec 16;13:1089729. doi: 10.3389/fmicb.2022.1089729 (PMC9800619; doi:10.3389/fmicb.2022.1089729)
Supplement: Supplementary file 1 [file Data_Sheet_1.docx]

**Supplemental Materials**

1. **Ordinary Differential Equations**

The DSS-induced colitis is a complex biological process that involved a variety of proteins in multiple cells. Our model simplifies this process and selects part of the proteins involved, leading to indirect interactions between these proteins that cannot be described by the law of mass action. Therefore, we use the Hill equations to describe the indirect interactions, as shown in the following ordinary differential equations (ODEs). Among them, the productions and degradations of the proteins are depicted with the law of mass action.

| $\frac{d\left[ N \right]}{dt}=k_{0}\times\left[ N \right]+k_{1}\times\frac{\left[ \beta catenin \right]^{2}}{{j_{1}}^{2}+\left[ \beta catenin \right]^{2}}\times\left[ N \right]+k_{2}\times\frac{\left[ Yap \right]^{2}}{{j_{2}}^{2}+\left[ Yap \right]^{2}}\times\left[ N \right]$ $+k_{3}\times\frac{\left[ pSTAT3 \right]^{2}}{{j_{3}}^{2}+\left[ pSTAT3 \right]^{2}}\times\left[ N \right]$  $-\gamma_{1}\times\frac{\left[ IL1\beta\right]^{2}}{{j_{4}}^{2}+\left[ IL1\beta\right]^{2}}\times[N]-\gamma_{2}\times{[N]}^{2}$ | (1) |
| --- | --- |
| $\frac{d\left[ MIR31 \right]}{dt}=k_{sMIR}+k_{MIRp65}\times\frac{\left[ pp65 \right]^{2}}{{j_{MIRp65}}^{2}+\left[ pp65 \right]^{2}}+k_{MIRpST}\times\frac{\left[ pSTAT3 \right]^{2}}{{j_{MIRpST}}^{2}+\left[ pSTAT3 \right]^{2}}$  $-\gamma_{I17RMIR}\times\frac{p2\times\left[ MIR31 \right]}{j_{I17RMIR}+p2\times\left[ MIR31 \right]}\times\left[ IL17RA \right]-\gamma_{GpMIR}\times\frac{p1\times\left[ MIR31 \right]}{j_{GpMIR}+p1\times\left[ MIR31 \right]}\times\left[ Gp130 \right]$  $-\gamma_{AxiMIR}\times\frac{\left( p3\times\left[ MIR31 \right] \right)^{2}}{{j_{AxiMIR}}^{2}+\left( p3\times\left[ MIR31 \right] \right)^{2}}\times\left[ Axin1 \right]$  $-\gamma_{LatMIR}\times\frac{\left( p4\times\left[ MIR31 \right] \right)^{2}}{{j_{LatMIR}}^{2}+\left( p4\times\left[ MIR31 \right] \right)^{2}}\times\left[ Lats1 \right]-k_{dMIR}\times\left[ MIR31 \right]$ | (2) |
| $\frac{d\left[ TNF\alpha\right]}{dt}=k_{0TNF}\times\frac{\left[ DSS \right]^{4}}{{j_{0TNF}}^{4}+\left[ DSS \right]^{4}}+k_{TNFI17I17R}\times\frac{\left[ IL17\_IL17RA \right]}{j_{TNFI17I17R}+\left[ IL17\_IL17RA \right]}-k_{dTNF}\times\left[ TNF\alpha\right]$ | (3) |
| $\frac{d\left[ pSTAT3 \right]}{dt}=k_{pSTI6Gp}\times\frac{\left[ IL6\_Gp130 \right]}{j_{pSTI6Gp}+\left[ IL6\_Gp130 \right]}+k_{pSTI1\beta}\times\frac{\left[ IL1\beta\right]^{4}}{{j_{pSTI1\beta}}^{4}+\left[ IL1\beta\right]^{4}}-k_{dpST}\times\left[ pSTAT3 \right]$ | (4) |
| $\frac{d\left[ \beta catenin \right]}{dt}=k_{s\beta ca}-\gamma_{\beta caYap}\times\frac{\left[ Yap \right]^{2}}{{j_{\beta caYap}}^{2}+\left[ Yap \right]^{2}}\times\left[ \beta catenin \right]$ $-\gamma_{\beta caAxi}\times\frac{\left[ Axin1 \right]^{2}}{{j_{\beta caAxi}}^{2}+\left[ Axin1 \right]^{2}}\times\left[ \beta catenin \right]$  $-k_{d\beta ca}\times\left[ \beta catenin \right]$ | (5) |
| $\frac{d\left[ IL6 \right]}{dt}=k_{0I6}\times\frac{\left[ DSS \right]^{2}}{{j_{0I6}}^{2}+\left[ DSS \right]^{2}}+k_{I6p65}\times\frac{\left[ pp65 \right]^{3}}{{j_{I6p65}}^{3}+\left[ pp65 \right]^{3}}-\gamma_{I6Gp1}\times\left[ IL6 \right]\times\left[ Gp130 \right]$ $+\gamma_{I6Gp2}\times\left[ IL6\_Gp130 \right]$  $-k_{dI6}\times\left[ IL6 \right]$ | (6) |
| $\frac{d\left[ IL17 \right]}{dt}=k_{I17pST}\times\frac{\left[ pSTAT3 \right]}{j_{I17pST}+\left[ pSTAT3 \right]}+k_{I17p65}\times\frac{\left[ pp65 \right]^{2}}{{j_{I17p65}}^{2}+\left[ pp65 \right]^{2}}$ $-\gamma_{I17I17R1}\times\left[ IL17 \right]\times\left[ IL17RA \right]$  $+\gamma_{I17I17R2}\times\left[ IL17\_IL17RA \right]-k_{dI17}\times\left[ IL17 \right]$ | (7) |
| $\frac{d\left[ IL1\beta\right]}{dt}=k_{0I1\beta}\times\frac{\left[ DSS \right]^{4}}{{j_{0I1\beta}}^{4}+\left[ DSS \right]^{4}}+k_{I1\beta p65}\times\frac{\left[ pp65 \right]^{2}}{{j_{I1\beta p65}}^{2}+\left[ pp65 \right]^{2}}$ $+k_{I1\beta I17I17R}\times\frac{\left[ IL17\_IL17RA \right]}{j_{I1\beta I17I17R}+\left[ IL17\_IL17RA \right]}$  $-k_{dI1\beta}\times\left[ IL1\beta\right]$ | (8) |
| $\frac{d\left[ Axin1 \right]}{dt}=k_{sAxi}-\gamma_{AxiMIR}\times\frac{\left( p3\times\left[ MIR31 \right] \right)^{2}}{{j_{AxiMIR}}^{2}+\left( p3\times\left[ MIR31 \right] \right)^{2}}\times\left[ Axin1 \right]-k_{dAxi}\times\left[ Axin1 \right]$ | (9) |
| $\frac{d\left[ Lats1 \right]}{dt}=k_{sLat}-\gamma_{LatMIR}\times\frac{\left( p4\times\left[ MIR31 \right] \right)^{2}}{{j_{LatMIR}}^{2}+\left( p4\times\left[ MIR31 \right] \right)^{2}}\times\left[ Lats1 \right]-k_{dLat}\times\left[ Lats1 \right]$ | (10) |
| $\frac{d\left[ Yap \right]}{dt}=k_{sYap}-k_{YapLat}\times\frac{\left[ Lats1 \right]^{2}}{{j_{YapLat}}^{2}+\left[ Lats1 \right]^{2}}\times\left[ Yap \right]-k_{dYap}\times\left[ Yap \right]$ | (11) |
| $\frac{d\left[ pp65 \right]}{dt}=k_{p65TNF}\times\frac{\left[ TNF\alpha\right]^{5}}{{j_{p65TNF}}^{5}+\left[ TNF\alpha\right]^{5}}+k_{p65I17I17R}\times\frac{\left[ IL17\_IL17RA \right]}{j_{p65I17I17R}+\left[ IL17\_IL17RA \right]}-k_{dp65}\times\left[ pp65 \right]$ | (12) |
| $\frac{d\left[ DSS \right]}{dt}=-k_{dDSS}\times\left( \left[ DSS \right]-\sum_{0}^{5\times24} \delta\left( T-{i\times T}_{0} \right)\times D_{0} \right)$ | (13) |
| $\frac{d\left[ IL6\_Gp130 \right]}{dt}=\gamma_{I6Gp1}\times\left[ IL6 \right]\times\left[ Gp130 \right]-\gamma_{dI6Gp2}\times\left[ IL6\_Gp130 \right]$ | (14) |
| $\frac{d\left[ Gp130 \right]}{dt}=k_{sGp}\times\frac{\left[ IL6 \right]^{3}}{{j_{sGp}}^{3}+\left[ IL6 \right]^{3}}-k_{dGp}\times\left[ Gp130 \right]-\gamma_{I6Gp1}\times\left[ IL6 \right]\times\left[ Gp130 \right]+\gamma_{I6Gp2}\times\left[ IL6\_Gp130 \right]$  $-\gamma_{GpMIR}\times\frac{p1\times\left[ MIR31 \right]}{j_{GpMIR}+p1\times\left[ MIR31 \right]}\times\left[ Gp130 \right]$ | (15) |
| $\frac{d[IL17\_IL17RA]}{dt}=\gamma_{I17I17R1}\times\left[ IL17 \right]\times\left[ IL17RA \right]-\gamma_{dI17I17R2}\times\left[ IL17\_IL17RA \right]$ | (16) |
| $\frac{d\left[ IL17RA \right]}{dt}=k_{sI17R}\times\frac{\left[ IL17RA \right]}{j_{sI17R}+\left[ IL17RA \right]}-k_{dI17R}\times\left[ IL17RA \right]-\gamma_{I17I17R1}\times\left[ IL17 \right]\times\left[ IL17RA \right]$  $+\gamma_{I17I17R2}\times\left[ IL17\_IL17RA \right]-\gamma_{I17RMIR}\times\frac{p2\times\left[ MIR \right]}{j_{I17RMIR}+p2\times\left[ MIR \right]}\times\left[ IL17RA \right]$ | (17) |

1. **The binding probabilities of MIR31 to four proteins**

MiRNA inhibits mRNA translation or degrades mRNA through complementary pairing with the mRNA of target protein ([Kim and Nam, 2006](#_ENREF_4), [Huntzinger and Izaurralde, 2011](#_ENREF_3)). The target mRNA will be degraded when complete complementary pairing occurs ([Baumberger and Baulcombe, 2005](#_ENREF_1), [Mi et al., 2008](#_ENREF_5)), while the target mRNA translation will be inhibited when the degree of complementary pairing is low ([Carthew and Sontheimer, 2009](#_ENREF_2)). In DSS-induced colitis, MIR31 inhibits the expression of IL17RA and Gp130 (mRNA translation) by complementation with the mRNAs of *Il17ra* and *Il6st*, thus inhibiting the occurrence of inflammation. In addition, MIR31 also promotes epithelial regeneration by complementing mRNAs of *Axin1* and *Lats2* to inhibit Axin1 and Lats1/2 expression (mRNA translation). However, our model ignores the complementary pairing processes with the above four mRNAs, and directly gives the final result that MIR31 inhibits the expressions of the four proteins by binding to them. Moreover, the probabilities of such binding inhibitions are randomly selected, which correlate to the protein concentrations in the model. The evolution equation of probability with time is given by

$$\left( \begin{aligned} \begin{aligned} p_{0}\left( t+dt \right) \\ p_{1}\left( t+dt \right) \\ p_{2}\left( t+dt \right) \end{aligned} \\ p_{3}\left( t+dt \right) \\ p_{4}\left( t+dt \right) \end{aligned} \right)=P\left( \begin{aligned} \begin{aligned} \begin{aligned} p_{0}\left( t \right) \\ p_{1}\left( t \right) \end{aligned} \\ p_{2}\left( t \right) \end{aligned} \\ p_{3}\left( t \right) \\ p_{4}(t) \end{aligned} \right)$$

where *t* is time with time step *dt*, and P represents the transition matrix. $p_{0}$ denotes the probability of MIR31 in a resting state without any binding. $p_{1}$, $p_{2}$, $p_{3}$ and $p_{4}$ represent the probability that MIR31 binding to Gp130, IL17RA, Axin1 and Lats1/2, respectively, with the constraint condition $p_{1}+p_{2}+p_{3}+p_{4}=1-p_{0}$. According to the binding dynamics, the transition matrix is given by

$$P=\left( \begin{matrix} {1-\rho}_{1}\times\left[ Gp130 \right]\times dt-\rho_{2}\times\left[ IL17RA \right]\times dt-\rho_{3}\times\left[ Axin1 \right]\times dt-\rho_{4}\times\left[ Lats1 \right]\times dt & d_{1}\times dt & d_{2}\times dt & d_{3}\times dt & d_{4}\times dt \\ \rho_{1}\times\left[ Gp130 \right]\times dt & {1-d}_{1}\times dt & 0 & 0 & 0 \\ \rho_{2}\times\left[ IL17RA \right]\times dt & 0 & {1-d}_{2}\times dt & 0 & 0 \\ \rho_{3}\times\left[ Axin1 \right]\times dt & 0 & 0 & {1-d}_{3}\times dt & 0 \\ \rho_{4}\times\left[ Lats1 \right]\times dt & 0 & 0 & 0 & {1-d}_{4}\times dt \end{matrix} \right)$$

where *ρ_i_* is the binding rate, and *d_i_* denotes the unbinding rate. As a result, the dynamical equations for MIR31 binding probabilities are given below according to the constraint condition.

| $\frac{dp_{0}}{dt}=\left( -\rho_{1}\times\left[ Gp130 \right]-\rho_{2}\times\left[ IL17RA \right]-\rho_{3}\times\left[ Axin1 \right]-\rho_{4}\times\left[ Lats1 \right] \right)\times p0$ $+d_{1}\times p1+d_{2}\times p2$  $+d_{3}\times p3+d_{4}\times p4$ | (18) |
| --- | --- |
| $\frac{dp_{1}}{dt}=\rho_{1}\times\left[ Gp130 \right]\times p0-d_{1}\times p1$ | (19) |
| $\frac{dp_{2}}{dt}=\rho_{2}\times\left[ IL17RA \right]\times p0-d_{2}\times p2$ | (20) |
| $\frac{dp_{3}}{dt}=\rho_{3}\times\left[ Axin1 \right]\times p0-d_{3}\times p3$ | (21) |
| $\frac{dp_{4}}{dt}=\rho_{4}\times\left[ Lats1 \right]\times p0-d_{4}\times p4$ | (22) |

**References**

BAUMBERGER, N. & BAULCOMBE, D. C. 2005. Arabidopsis ARGONAUTE1 is an RNA Slicer that selectively recruits microRNAs and short interfering RNAs. *Proceedings of the National Academy of Sciences,* 102**,** 11928-11933.

CARTHEW, R. W. & SONTHEIMER, E. J. 2009. Origins and Mechanisms of miRNAs and siRNAs. *Cell,* 136**,** 642-55.

HUNTZINGER, E. & IZAURRALDE, E. 2011. Gene silencing by microRNAs: contributions of translational repression and mRNA decay. *Nat Rev Genet,* 12**,** 99-110.

KIM, V. N. & NAM, J.-W. 2006. Genomics of microRNA. *Trends in Genetics,* 22**,** 165-173.

MI, S., CAI, T., HU, Y., CHEN, Y., HODGES, E., NI, F., WU, L., LI, S., ZHOU, H., LONG, C., CHEN, S., HANNON, G. J. & QI, Y. 2008. Sorting of small RNAs into Arabidopsis argonaute complexes is directed by the 5' terminal nucleotide. *Cell,* 133**,** 116-27.

1. **Fitting of the model parameters**

In our model, Pearson correlation coefficient is used to measure the correlation between experimental and simulation results:

$$R=\frac{\sum_{i=1}^{n} (Y_{exp i}-\overline{Y_{\exp}})(Y_{\mathrm{simi}}-\overline{Y_{\mathrm{sim}}})}{\sqrt{\sum_{i=1}^{n} {(Y_{exp i}-\overline{Y_{\exp}})}^{2}}\sqrt{\sum_{i=1}^{n} {(Y_{\mathrm{simi}}-\overline{Y_{\mathrm{sim}}})}^{2}}}$$

where $Y_{\mathrm{expi}}$ is the experimental data and $Y_{\mathrm{simi}}$ is the corresponding simulation results. $\overline{Y_{\exp}}$ and $\overline{Y_{\mathrm{sim}}}$ are the average values of the experimental and simulation results, respectively. As a result, compared with the experimental results in our previous paper and new experiments, the Pearson correlations are 0.93, 0.88, 0.86 and 0.90 for time-dependent responses of the relative levels of MIR31, p-p65, p-STAT3 and cell number, respectively.

**Table S1: Variables of the model**

| **Variable** | **Description** | **Initial Values** |
| --- | --- | --- |
| **[DSS]** | external stimulus | 0.35% w/v |
| **[N]**  **[MIR31]**  **[TNFα]**  **[pSTAT3]**  **[βcatenin]**  **[IL6]**  **[IL17]**  **[IL1β]**  **[Axin1]**  **[Lats1]**  **[Yap]**  **[pp65]**  **p_0_**  **p_1_**  **p_2_**  **p_3_**  **p_4_**  **[IL6_Gp130]**  **[IL17_I17RA]**  **[Gp130]**  **[IL17RA]** | Proliferative cell number  Concentration of MIR31  Concentration of TNFα  Concentration of p-STAT3  Concentration of β-catenin  Concentration of IL6  Concentration of IL17  Concentration of IL1β  Concentration of Axin1  Concentration of Lats1/2  Concentration of Yap  Concentration of p-p65  Probability that MIR31 is inactive  Probability that MIR31 binding to Gp130  Probability that MIR31 binding to IL17RA  Probability that MIR31 binding to Axin1  Probability that MIR31 binding to Lats1/2  Concentration of IL6 and Gp130 complexus  Concentration of IL17 and IL17RA complexus  Concentration of IL6 receptor  Concentration of IL17 receptor | 3000  1.013  0  0  1.0  0  0  0  1.0  1.0  1.0  0  1  0  0  0  0  0  0  0  0 |

**Table S2: Parameters of the model**

| **Variable** | **Description** | **Values** |
| --- | --- | --- |
| **k_0_**  **k_1_**  **k_2_**  **k_3_**  **j_1_**  **j_2_**  **j_3_**  **j_4_**  **γ_1_**  **γ_2_** | Cell proliferation rate  Cell proliferation rate by β-catenin  Cell proliferation rate by Yap  Cell proliferation rate by p-STAT3  Michaelis constant of β-catenin-dependent cell proliferation  Michaelis constant of Yap-dependent cell proliferation  Michaelis constant of p-STAT3-dependent cell proliferation  Michaelis constant of IL1β-dependent cell degradation  Cell degradation rate by IL1β  Cell degradation rate | 0.0039  0.001  0.001  0.00088  0.5  0.8  1.0  5.3  0.0091  0.0000013 |
| **k_sMIR_**  **k_dMIR_**  **k_MIRp65_**  **k_MIRpST_**  **γ_I17RMIR_**  **γ_GpMIR_**  **γ_AxiMIR_**  **γ_LatMIR_**  **j_MIRp65_**  **j_MIRpST_**  **j_I17RMIR_**  **j_GpMIR_**  **j_AxiMIR_**  **j_LatMIR_** | Production rate of MIR31  Degradation of MIR31  Production rate of MIR31 by p-p65  Production rate of MIR31 by p-STAT3  Inhibition rate of MIR31 production by IL17RA  Inhibition rate of MIR31 production by Gp130  Inhibition rate of MIR31 production by Axin1  Inhibition rate of MIR31 production by Lats1/2  Michaelis constant of p-p65-dependent MIR31 production  Michaelis constant of p-STAT3-dependent MIR31 production  Michaelis constant of IL17RA-dependent MIR31 production  Michaelis constant of Gp130-dependent MIR31 production  Michaelis constant of Axin1-dependent MIR31 production  Michaelis constant of Lats1/2-dependent MIR31 production | 0.01013  0.01  0.18  0.2  0.008  0.003  0.015  0.02  2.1  0.7  6  6  7  6 |
| **k_0TNF_**  **k_TNFI17I17R_k_dTNF_**  **j_0TNF_**  **j_TNFI17I17R_** | Production rate of TNFα by DSS  Production rate of TNFα by IL17_IL17RA  Degradation rate of TNFα  Michaelis constant of DSS-dependent TNFα production  Michaelis constant of IL17_IL17RA-dependent TNFα production | 0.02  0.002  0.002  1.0  0.1 |
| **k_pSTI6Gp_**  **k_pSTI1β_**  **k_dpST_j_pSTI6Gp_**  **j_pSTI1β_** | Production rate of p-STAT3 by IL6_Gp130  Production rate of p-STAT3 by IL1β  Degradation rate of p-STAT3  Michaelis constant of IL6_Gp130-dependent p-STAT3 production  Michaelis constant of IL1β-dependent p-STAT3 production | 0.003  0.001  0.0018  1.0  6.5 |
| **k_sβca_**  **γ_βcaYap_**  **γ_βcaAxi_k_dβca_**  **j_βcaYap_**  **j_βcaAxi_** | Production rate of β-catenin  Inhibition rate of βcatenin by Yap  Inhibition rate of βcatenin by Axin1  Degradation rate of β-catenin  Michaelis constant of Yap-dependent inhibition to β-catenin  Michaelis constant of Axin1-dependent inhibition to β-catenin | 0.001  0.001  0.01  0.001  0.2  0.2 |
| **k_0I6_**  **k_I6p65_**  **γ_I6Gp1_**  **k_dI6_**  **j_0I6_**  **j_I6p65_** | Production rate of IL6 by DSS  Production rate of IL6 by p-p65  Consumption rate of IL6 to Gp130  Degradation rate of IL6  Michaelis constant of DSS-dependent IL6 production  Michaelis constant of p-p65-dependent IL6 production | 0.006  0.004  0.001  0.002  1.5  2.5 |
| **k_I17pST_k_I17p65_**  **γ_I17I17R1_k_dI17_**  **j_I17pST_**  **j_I17p65_** | Production rate of IL17 by p-STAT3  Production rate of IL17 by p-p65  Consumption rate of IL17 to IL17RA  Degradation rate of IL17  Michaelis constant of p-STAT3-dependent IL17 production  Michaelis constant of p-p65-dependent IL17 production | 0.004  0.002  0.001  0.004  1.5  3.5 |
| **k_0I1β_**  **k_I1βp65_**  **k_I1βI17I17R_**  **k_dI1β_**  **j_0I1β_**  **j_I1βp65_**  **j_I1βI17I17R_** | Production rate of IL1β by DSS  Production rate of IL1β by p-p65  Production rate of IL1β by IL17_IL17RA  Degradation rate of IL1β  Michaelis constant of DSS-dependent IL1β production  Michaelis constant of p-p65-dependent IL1β production  Michaelis constant of IL17_IL17RA-dependent IL1β production | 0.01  0.01  0.006  0.002  0.7  2.0  0.15 |
| **k_sAxi_**  **k_dAxi_** | Production rate of Axin1  Degradation rate of Axin1 | 0.001  0.001 |
| **k_dDSS_** | Degradation rate of DSS | 0.035/40 |
| **k_sLat_**  **k_dLat_** | Production rate of Lats1/2  Degradation rate of Lats1/2 | 0.001  0.001 |
| **k_sYap_**  **k_YapLat_**  **j_YapLat_**  **k_dYap_** | Production rate of Yap  Lats1/2-dependent production rate of Yap  Michaelis constant of Lats1/2-dependent inhibition to Yap  Degradation rate of Yap | 0.001  0.001  0.2  0.001 |
| **k_p65TNF_**  **k_p65I17I17R_**  **j_p65TNF_**  **j_p65I17I17R_**  **k_dp65_** | TNFα-dependent production rate of p-p65  IL17_IL17RA-dependent production rate of p-p65  Michaelis constant of TNFα-dependent inhibition to p-p65  Michaelis constant of IL17I_L17RA-dependent inhibition to p-p65  Degradation rate of p-p65 | 0.005  0.004  1.7  0.15  0.002 |
| **γ_I6Gp2_** | Dissociation rate of IL6_Gp130 | 0.002 |
| **γ_I17I17R2_** | Dissociation rate of IL17_IL17RA | 0.005 |
| **k_sGp_**  **k_dGp_**  **j_sGp_** | IL6-dependent production rate of Gp130  Degradation rate of Gp130  Michaelis constant of IL6-dependent Gp130 production | 0.003  0.003  0.003 |
| **k_sI17R_**  **k_dI17R_**  **j_sI17R_** | IL17-dependent production rate of IL17RA  Degradation rate of IL17RA  Michaelis constant of IL17-dependent IL17RA production | 0.004  0.003  0.004 |
| **ρ_1_, ρ_2_, ρ_3_, ρ_4_**  **d_1_,d_2_ , d_3_, d_4_** | Probability combining to MIR31 of Gp130,IL17RA,Axin1,Lats1/2  Probability disassociating from MIR31 of Gp130,IL17RA,Axin1,Lats1/2 | 0.05  0.0008 |
